# The Physiopathology of Cardiorenal Syndrome: A Review of the Potential Contributions of Inflammation

**DOI:** 10.3390/jcdd4040021

**Published:** 2017-11-29

**Authors:** John G. Kingma, Denys Simard, Jacques R. Rouleau, Benoit Drolet, Chantale Simard

**Affiliations:** 1 Department of Medicine, Faculty of Medicine, Pavillon Ferdinand Vandry, 1050, Avenue de la Médecine, Université Laval, Quebec, QC G1V 0A6, Canada; Jacques-r.rouleau@fmed.ulaval.ca; 2 Centre de Recherche de l’Institut Universitaire de Cardiologie et de Pneumologie de Québec–Université Laval, 2725, Chemin Sainte-Foy, Quebec, QC G1V 4G5, Canada; denis.simard@criucpq.ulaval.ca (D.S.); benoit.drolet@pha.ulaval.ca (B.D.); chantale.simard@pha.ulaval.ca (C.S.); 3 Faculty of Pharmacy, Pavillon Ferdinand Vandry, 1050, Avenue de la Médecine, Université Laval, Quebec, QC G1V 0A6, Canada

**Keywords:** cardiorenal syndrome, inflammation, epigenetics, heart dysfunction, kidney dysfunction, organ crosstalk

## Abstract

Inter-organ crosstalk plays an essential role in the physiological homeostasis of the heart and other organs, and requires a complex interaction between a host of cellular, molecular, and neural factors. Derangements in these interactions can initiate multi-organ dysfunction. This is the case, for instance, in the heart or kidneys where a pathological alteration in one organ can unfavorably affect function in another distant organ; attention is currently being paid to understanding the physiopathological consequences of kidney dysfunction on cardiac performance that lead to cardiorenal syndrome. Different cardiorenal connectors (renin–angiotensin or sympathetic nervous system activation, inflammation, uremia, etc.) and non-traditional risk factors potentially contribute to multi-organ failure. Of these, inflammation may be crucial as inflammatory cells contribute to over-production of eicosanoids and lipid second messengers that activate intracellular signaling pathways involved in pathogenesis. Indeed, inflammation biomarkers are often elevated in patients with cardiac or renal dysfunction. Epigenetics, a dynamic process that regulates gene expression and function, is also recognized as an important player in single-organ disease. Principal epigenetic modifications occur at the level of DNA (i.e., methylation) and histone proteins; aberrant DNA methylation is associated with pathogenesis of organ dysfunction through a number of mechanisms (inflammation, nitric oxide bioavailability, endothelin, etc.). Herein, we focus on the potential contribution of inflammation in pathogenesis of cardiorenal syndrome.

## 1. Introduction

Heart and kidney function are strongly connected. Organ crosstalk, or biological communication between organs, involves cellular, molecular and neural factors; it plays an essential role in physiological homeostasis and in the event of malfunction can induce organ dysfunction [1,2,3,4,5]. Multi-factorial mechanisms that lead to cardiorenal syndrome (i.e., a pathophysiological disorder of the heart and kidneys where acute or chronic failure in one organ adversely affects the other [6]) are not only limited to hemodynamic parameters (extracellular fluid volume, cardiac output, arterial pressure, etc.) [7]. Cardiorenal syndrome involves complex interactions at the molecular level which induce vessel inflammation, atherosclerosis, cardiac fibrosis, and hypertrophy [8]; in addition, structural and biochemical abnormalities can adversely affect cardiovascular or renal function [9,10]. However, these complex associations could result from various confounding factors and not direct organ-to-organ interactions. Specific cardiorenal connectors (higher renin–angotensin system activity, oxidative stress, inflammation, increased sympathetic nervous system activity) have been studied [11]. Furthermore, non-traditional risk factors such as metabolic syndrome, anemia, arterial stiffness, chronic inflammation, and uremic toxins are also implicated [12]. To date, five different subtypes of cardiorenal syndrome, each reflecting primary as well as secondary pathologies, have been described [13]. In type 1 and 2 cardiorenal syndrome both acute and chronic heart failure produce AKI or accelerated chronic kidney disease (CKD) [14,15]. In types 3 and 4, both AKI and accelerated CKD are present and lead to heart failure. Type 5 involves systemic disorders, for example sepsis, whereby both organs are injured simultaneously [16].

The causal association between CKD and cardiovascular risk was initially deliberated by Bright in 1836 [17]; his observations have since been confirmed but the underlying physiopathological mechanisms remain unresolved. Why should we be interested in studying the physiopathological processes involved in inter-organ interactions such as the kidneys and the heart? 

Firstly, patients with CKD are amongst the highest risk groups for adverse cardiovascular events and cardiovascular-related mortality and therefore require particular clinical attention [18]. Additionally, the economic burden of cardiorenal syndrome in developed countries is increasing; estimates in the United States alone indicate that billions of dollars are spent annually on these patients primarily because of prolonged hospitalizations and long-term morbidities. Secondly, clinical and epidemiological observations support the strong causal association between CKD, cardiovascular risk, and heart failure [19,20]. This suggests that prevention of either component could help to attenuate disease progression and death [21,22]; however, it is clear that co-existence of either of these conditions significantly affects patient outcomes [23]. Of note is that renal failure does not by itself cause death [24]; cardiogenic or non-cardiogenic injury is believed to be responsible for the high mortality in these patients [25,26]. 

Recent findings document that detrimental bi-directional interaction between different renocardiac connectors influence vascular endothelium (renin–angiotensin–aldosterone system, sympathetic nervous system, activated inflammatory mediators, reactive oxygen species, endothelin and uremic toxins) and contribute to progression of multi-organ failure [7,27]. Under normal circumstances, bi-directional communications between the heart and kidneys coordinate to modulate cardiac output, vascular tone, and volume status as well as excretion of metabolic waste products. Disruption of either of these pathways contributes to progressive cardiovascular or kidney dysfunction; indeed, failure of one organ system appears to accelerate structural damage and failure of another organ [7]. Vascular endothelial health may also be key to reducing the negative effects of cardiorenal syndrome; activation of the endothelium is important for innate immunity and inflammation, complement activation, coagulation, platelet function, and vasoconstriction [28]. Capillary loss, induced by endothelial injury, can also lead to ischemia and its attendant complications. 

Inflammation is a potentially important stressor for acute and chronic cardiorenal dysfunction. Altered endothelial regulation [29], which may be linked to inflammation, has been documented to affect afterload–preload mismatch in heart failure patients consequent to greater arterial stiffness or reduced venous capacitance and increased venous pressure [30]. Higher central venous pressures (i.e., including higher renal venous pressures) promote kidney dysfunction, injury, and release of pro-inflammatory cytokines [31,32]. Clearly, physiopathological consequences and how they influence organ crosstalk and pathogenesis remain complex.

In this review, we discuss clinical and experimental findings related to the role of inflammation in the pathogenesis of cardiorenal syndrome as well as heart and kidney disease. Herein we attempt to establish a role for inflammation in the physiopathology of cardiorenal syndrome per se without taking into account the multifaceted presentations of CKD from early kidney involvement through renal replacement therapy and organ transplantation. Clinical and basic science reports were searched using MEDLINE, PubMed, and Google Scholar with the keywords cardiorenal syndrome, heart–kidney interactions, ischemia, heart and kidney function, inflammation, and various combinations thereof. Finally, experimental data from our own studies in this field were also consulted.

## 2. Kidney Pathology

The pathogenesis of kidney disease is complicated by the cellular composition and anatomy of nephrons; injured tubular epithelial cells contribute to functional impairment of water and electrolyte homeostasis along with reduced disposal of metabolic waste products. Enhanced inflammatory status also contributes to renal injury and reduced kidney function [7,33,34]. Furthermore, worsening kidney function directly affects acute/chronic cardiac disease [35,36] and could contribute to poor clinical outcomes [37]. 

Various triggers (i.e., focal segmental glomerulosclerosis, mesangial proliferation, tubular necrosis, or interstitial fibrosis, etc.) produce pathologically distinct lesions in the kidneys [38]. Renal vessel injury causes intravascular clotting and increased risk of extra-vascular hemorrhage; a close relation exists between clotting and inflammation due to activation of platelets (with attendant aggregation) that stimulates release of cytokines and chemokines to promote leukocyte recruitment [39,40]. A coagulation-mediated release of mitogenic factors (epidermal growth factors, cytokines, chemokines, etc.) promotes re-epithelialization of the epithelial cell barrier, thereby limiting fluid losses [38]. Persistent de-epithelialization increases the risk of chronic injury; however, uncoordinated epithelial hyperplasia is also detrimental (i.e., glomerular crescent formation) [41]. Moreover, increased attention is being directed towards the role of miRNAs in epithelial regeneration [42,43]. Inflammatory markers such as C-reactive protein could be associated with a higher risk in cardiovascular mortality in patients with CKD [44]; however, questions remain regarding potential synergy [45]. 

Stimulation of inflammation mechanisms during AKI results in release of soluble mediators into the bloodstream that exert harmful effects on distant organs. Knowledge on physiopathological mechanisms is limited; however, insights into cellular and molecular mechanisms are currently being addressed in pre-clinical studies. For instance, after organ injury, monocytes (involved in both tissue damage and repair) release cytokines into the peripheral circulation that trigger pathogenic mechanisms in distant organs [46]. Monocyte migration with the resultant release of pro-inflammatory cytokines has been documented in type 1 cardiorenal syndrome [47]; interleukin levels (IL-6, IL-8, etc.) are also substantially elevated [48]. A recent report on inflammatory mediators and their role in the different types of cardiorenal syndrome is available [2].

A sophisticated connection or crosstalk exists between the sympathetic nervous and immune systems; activation of the sympathetic nervous system amplifies the release of inflammatory cells (monocytes, leukocytes, etc.) into the bloodstream, resulting in low-grade systemic inflammation [49] and can affect cardiovascular regulation. 

Neuroimmunomodulation interventions that are based on bidirectional control by the brain of physiological functions of various organs (heart, intestines, lung, immune system, etc.) are also being tested [50]. Recent clinical findings using catheter-based renal denervation documented significant modifications in inflammatory status of monocytes in hypertensive patients when the activity of the sympathetic nervous system was markedly reduced [51]. Similar findings were reported with experimental hypertension in mice [52]. In addition, Tang and co-workers recently reported an increased risk of ventricular fibrillation in dogs with CKD, which was attenuated by renal denervation [53]. These results were attributed to reduced left ventricle (LV) hypertrophy, sympathetic activation, and inflammation. Early clinical trials with vagus nerve stimulation also document substantial attenuation of inflammation; however, further studies are required to identify principal neural circuits that regulate kidney function [54]. The reader is referred to a recent review that examined innervation of kidney and potential cardiovascular control in pathological conditions [55]. A disequilibrium between pro- and anti-inflammatory cytokines following activation of inflammatory cells is accompanied by oxidative stress [56,57]; these findings support the concept for a mechanistic link between hypertension and atherosclerosis, which can be targeted therapeutically. 

## 3. Cardiac Pathology

Coronary artery disease most often results in an acute coronary occlusive event due to rupture of atherosclerotic plaque; various inflammatory cells contribute to atherogenic pathogenesis and its complications [58] (the reader is referred to a recent review article by Sager and Nahrendorf for details [59]). 

Myocardial tissue is highly dependent on delivery of oxygen and nutrients in blood, therefore any interruption in blood flow results in a marked disruption of cardiac function. Physiopathology of acute myocardial infarction is well established [60,61,62], with two types of ischemic injury: (1) reversible, where myocytes survive periods of ischemia <15 min duration; and (2) irreversible, with no capacity for myocyte recovery. In irreversible injury, marked cellular ultrastructural changes (i.e., cell swelling, denaturation of intracellular proteins, membrane disruption, etc.) are produced due to metabolic failure and rapid depletion of high-energy stores [63,64,65]. 

Inflammatory cells also participate in myocardial injury, as well as post-ischemic repair of affected myocardium [66]. For instance, cardiac remodeling, which is associated with increased risk of heart failure, ischemic heart disease, and sudden death is commonly observed in early CKD patients [67,68]. In rats with CKD, cardiac interstitial fibrosis increases without affecting LV function; however, expansion of renal tubules (in medullar sections) along with interstitial fibrosis and inflammation has also been reported [69,70]. Additional studies in rats using gene chip array analysis were unable to show augmented cell death (apoptosis) in cardiac sections from rats with CKD [13]; on the basis of these findings they suggested that CKD produces pathological changes in the heart that are not related to myocyte death. Cardiomyopathy resulting from viral, bacterial, or non-infectious (autoimmune, hypersensitivity, etc.) causes is also associated with impaired myocardial function [71]. Inflammatory signaling networks also link different organs and systems to atherosclerotic plaque [66]. 

## 4. Inflammation

Inflammation is a non-traditional risk factor in cardiovascular disease; in addition, it is a systemic condition mediated by multiple factors (cytokines, complement, etc.). Ischemia, infection, and uremic milieu have the potential to stimulate diverse inflammatory components in both kidney and heart disease [72]. Leukocytosis and C-reactive protein are known to be independently related to cardiovascular problems outcomes [73,74,75]. An association between inflammatory markers and outcomes has also been documented in CKD patients [76,77]; inflammation may therefore be a crucial link for increased cardiovascular risk in the setting of kidney disease. Direct contribution of immune- or inflammation-mediated damage to pathogenesis of cardio-renal syndrome needs to be confirmed [78]. 

Under adverse conditions such as myocardial ischemia, different inflammatory cells, platelets, and fibroblasts contribute to chronic production of eicosanoids and other lipid second messengers; this is potentially deleterious and can result in dysfunctional excitation–contraction coupling, bioenergetic inefficiency, and accelerated necrosis, all of which contribute to myocardial pathology. We refer the reader to the review paper by Jenkins et al. that examined the role of myocardial eicosanoid signaling pathways in cardiac function [79]. Arachidonic acid (AA) metabolites are crucial for regulation of normal myocardial function; cardiac AA metabolism is coordinated through paracrine signaling to regulate blood flow, contraction, and hemodynamic function. Eicosanoids resulting from activation of intracellular phospholipases are oxidized by: (1) cyclooxygenases (COXs); (2) lipoxygenases (LOXs); and (3) cytochrome P450 (CYP450) enzymes to produce lipid second messengers that regulate cellular processes (i.e., gene transcription, ion channel kinetics, etc.). 

Myocardial membranes are highly enriched with choline and ethanolamine plasmalogens that comprise esterified AA at the *sn-2* position; these plasmalogens markedly affect membrane dynamics [80,81,82]. Phospholipase A_2_ catalyzes hydrolysis of the *sn-2* ester linkage of glycerophospholipids to generate free fatty acids such as AA and other lysolipid products. The important role of myocardial phospholipase A_2_-activated release of AA from cellular phospholipids is well established [83,84,85]. During ischemia, myocardial phospholipase modulation affects membrane-associated protein complexes, thereby leading to altered metabolism and bioenergetics.

COXs catalyze AA to form prostaglandin G_2_; COX-1 is expressed constitutively and is involved in cardiac homeostasis [86]. On the other hand, COX-2 can mediate inflammation and exacerbate cardiac function [87,88] but it has also been suggested to play a role in cardioprotection [89,90]. Further studies are necessary to assess the potential influence of eicosanoid mediated signaling in physiopathology of organ failure. 

Catalysis of AA by LOXs to hydroperoxyeicosatetraenoic acids results in production of hydroxyeicosatrienoic (HETE) derivatives. In normal myocardium HETEs contribute to regulation of cardiac function; post-ischemic activation of lipoxygenases may be important in the development of cardiac fibrosis and hypertrophy [91]. Upregulation of LOX products may also contribute to myocyte apoptosis [79]. The role of LOX-derived products in cardio-renal interactions and physiopathology requires further investigation; indeed, the role of specific HETE moieties remains to be established.

In cardiac tissues, several CYP450-generated eicosanoids have been identified and linked to the pathogenesis of hypertension, myocardial infarction, AKI and stroke [92,93,94,95,96,97]. The CYP epoxygenases (CYP2C, CYP2J) convert AA to epoxyeicosatrienoic acids (EETs), while the ω-hydroxylases (CYP4A, CYP4F) generate ω-hydroxyeicosatetraenoic acids (HETEs) [98,99]. EET regioisomers have potent vasodilator and anti-inflammatory properties [95]; these EETs are rapidly broken down by soluble epoxide hydrolases to dihydroxyeicosatrienoic acids (DHETs) and modulate vascular responses to endogenous endothelium-active compounds such as acetylcholine, bradykinin, nitric oxide, etc. Arachidonic acid can also be metabolized by either CYP4A or CYP4F to 20-hydroxyeicosatetraenoic acid (20-HETE), an autocrine factor that promotes vasoconstriction and inflammation and regulates renal blood flow and afferent arteriolar function [100,101]. Functional polymorphisms of CYP epoxygenases and ω-hydroxylases are implicated in cardiovascular disease in genetic epidemiology studies in humans [102,103]. CYP450-metabolites are also involved in regulation of extracellular fluid volume as well as renal and peripheral vascular tone [93,104,105]; however, the functional relationship of these metabolites and vascular dysfunction and their contribution to pathogenesis of organ failure remains unclear. 

CYP450-derived AA metabolites have been evaluated in animal models of cardiac pathology [106,107]. The 20-HETE levels increase in cardiac hypertrophy and are associated with cardiotoxicity [79,106], but in kidney 20-HETE is associated with vasoconstriction of afferent arterioles [108,109]. The biological actions of HETEs remain to be established. Findings from our laboratory in dogs with kidney dysfunction (stage 2) reported a significant lowering of cardiac 20-HETE levels but no effect on other EET catabolites (i.e., 14,15-DHET) [110]. A similar change was not observed in other organs (i.e., liver, kidneys) from the same animals; expression and activity levels of CYP-derived metabolites were not studied but merit further investigation. 

In clinical studies, inflammation and its impact on cardiovascular renal pathogenesis remain to be established. Weiner et al. performed a study with two patient cohorts (Cardiovascular Health Study, Atherosclerosis in Communities Study) [45]; they documented an association between inflammation and increased risk of stroke, cardiac events, and death. On the other hand, other studies (Dallas Heart Study, Baltimore Longitudinal Study of Aging) have provided evidence for an association between inflammation and cardiovascular risk [111,112]. Direct evidence that inflammation is an important contributor to cardiorenal syndrome is incomplete at present; however, both chronic heart failure and CKD are considered states of chronic inflammation (with elevated levels of circulating inflammatory mediators). As such, inflammatory burden and duration may chronicle development of cardiorenal disease [113]. 

## 5. Epigenomics

Epigenetic inheritance is responsible for phenotypic differences between cell types in multicellular organisms; epigenetics is considered a dynamic process that regulates gene expression patterns, accessibility to the genome through gene expression and ultimately gene function [114]. In mammals, primary mechanisms of epigenetic modifications include DNA methylation and a combination of post-translational modifications of the histone proteins (involved in packaging chromatin) that affect chromatin structure and expression of genes (the reader is referred to a recent review by Napoli and colleagues [8] for additional details). Interestingly, noncoding RNAs such as miRNAs may regulate expression of drug-metabolizing genes [115,116]. For instance, regulation of pregnane X receptor (PXR; a transcription factor) by miRNA148a indirectly affects expression of CYP3A4 [117]. Various functions of different short and long non-protein coding RNAs remain to be determined; however, their overall importance in epigenetic regulation of gene expression is increasingly recognized. Within the context of the physiopathology of kidney and heart disease, epigenetic modifications occur particularly at the level of histone-modifying enzymes [8]. For instance, renal failure has been documented to increase cardiac histone H3 epigenetics [118]; these findings directly link renal failure with induction of cardiomyopathy-related genes. Other studies reported the occurrence of epigenetic modifications in single-organ disease. For example, aberrant DNA methylation contributes to accelerated atherosclerosis by upregulating/downregulating susceptible genes [119]; uremia can also affect DNA methylation, which suggests that epigenetic alterations are involved in cardiorenal syndrome. Homocysteine and S-adenosylmethionine levels in patients with vascular disease are increased and have been associated with DNA hypomethylation. In addition, inflammation has been proposed to link low homocysteine levels with cardiovascular outcomes in patients with CKD; inflammation also causes aberrant DNA methylation [120]. Evidence for histone modification has been derived from the use of histone deacetylase (HDAC) inhibitors that act as signal responsible repressors of cardiac hypertrophy by regulating specific transcription factors and cardiac gene expression [121,122]. Although HDAC inhibitors promote expression of protective genes, they could also block expression of pathological genes. An example in the heart for cardioprotection by HDAC inhibitors relates to their ability to de-repress expression of protective cardiac genes that code for endogenous antioxidant enzymes; these drugs also reverse the myosin isoform switch (associated with functional consequences of heart failure) [123,124].

Endothelin (ET-1) also contributes to cardiac and renal physiopathology; elevated ET-1 levels are found in arterial hypertension and chronic renal failure [125,126]. DNA methylation and histone modification patterns have been documented to affect transcriptional activity of *edn1*, an ET-1 encoding gene [127]. Another major player in post ischemic endothelial dysfunction is nitric oxide (NO). Epigenetic mechanisms that affect expression of various nitric oxide synthase isoforms contribute to bioavailability of NO and ultimately to the potential for tissue injury caused, in part, by impaired blood flow distribution in the heart and kidneys [128,129]. 

## 6. Conclusions

A better understanding of underlying mechanisms involved in organ crosstalk in relation to heart and kidney disease is required to improve clinical outcomes; the same is true for co-morbidities including cardiorenal syndrome. It may not be that important which organ is initially affected, but how pathogenesis in one organ affects other distant organs merits investigation. Inflammation may be a key underlying mechanism in the progression of multi-organ disease; however, epigenetics and their influence on inflammation and disease progression also warrant consideration. Controlled clinical studies with drugs investigating these factors in cardiorenal syndrome patients remain at a preliminary stage, however, results from ongoing studies will undoubtedly improve future therapeutic approaches for patients.
